# Mechanical Properties and Applications of Advanced Ceramics

**DOI:** 10.3390/ma17133143

**Published:** 2024-06-27

**Authors:** Lidija Ćurković, Irena Žmak

**Affiliations:** Faculty of Mechanical Engineering and Naval Architecture, University of Zagreb, 10000 Zagreb, Croatia

The development of new materials or technologies has created turning points throughout the history of mankind, as the societies that had access to this new knowledge were able to overpower others. Ceramic materials were the first man-made materials; they were formed from clay and hardened by pit firing thousands of years ago. Modern society strongly relies on ceramics, not only for enjoying our daily meals and drinks, but more importantly for their use in demanding and highly specialized applications, numerous industrial and household appliances, vehicles, computers, communication equipment, aerospace technology, etc. Advanced ceramics have found their way into many engineering fields where metals have failed due to characteristic properties of ceramic materials, such as their very high hardness (hence, high wear resistance), impressive chemical resistance (i.e., corrosion resistance in many aggressive media), and the ability to retain these favorable properties at the highest temperatures. Some advanced ceramics have a high electrical resistance while others are used for semiconductor applications. Often, but not always, ceramics have a low density. In addition to engineering, advanced ceramics are necessary for many biomedical applications, including artificial joints, implants, dental crowns, stylish veneers, etc.

Developing new ceramic materials, ceramic coatings, or ceramic composites, and optimizing and modeling the production technologies used to manufacture them, are important and complex research topics. The issue of the sustainability of ceramics has been tackled in many ways, including by selecting and optimizing the use of greener additives, developing more energy-efficient sintering techniques, and developing ways to reuse or recycle unfired ceramic waste which is inevitably created during production. As we continue to be faced with increasing amounts of end-of-life electric and electronic products, a number of concerns are being directed toward finding more environmentally friendly and technically adequate yet cost-effective technical ceramics, such as lead-free piezoelectric ceramics.

Our interest in advanced ceramics has been maintained for almost two decades, allowing us to continually learn and grow, fund young and enthusiastic doctoral students, cooperate, publish, fund new research, and invest in new laboratory equipment. The scope of this Special Issue entails a brief overview of the current research related to advanced ceramics, including the development of photocatalysis for wastewater, improving the properties of ceramics by producing composite materials, an estimation of the long-term properties of ceramics and the mathematical modeling of properties, an evaluation of wear resistance, and the possibilities of recycling advanced ceramics. The advanced ceramics addressed within this Issue include aluminum oxide, titanium oxide, zirconium oxide with added titanium nitride, lithium tantalite—a unique electronic material—and wear-resistant basalt-based glass ceramics.

Basalt-based glass ceramics, i.e., olivine–pyroxene basalt, have been proven to be applicable as key structural components within metallurgical and mining equipment as a replacement for metallic materials. These ceramics were found to be highly resistant to cavitation wear based on the tests performed to calculate the mass loss vs. the cavitation time, the surface degradation level, and changes in morphology. In contrast, raw basalt had a low resistance to cavitation wear. It was shown that the image analysis software ImageProPlus, together with standard mass loss measurements, is a reliable yet rapid testing method for selecting materials to be used within metallurgical and mining components [1].

The room-temperature creep resistance of a single crystal of LiTaO_3_ was successfully estimated using nanoindentation, which was confirmed to be a useful, rapid, and precise method for testing brittle materials and small products compared to standard creep measurements. Nanoindentation has been shown to induce creep deformation even under elastic deformations at room temperature. In the experiments, the holding depth increased almost linearly with the creep displacement and the creep rate. The size of the spherical tip of the nanoindenter applied seemed not to be correlated with creep displacement and the creep rate. When the holding strain was increased, the total creep strain increased accordingly, while the strain rate of steady-state creep declined. Additionally, a nanoindentation size effect was observed, i.e., a smaller indenter tip produced a larger amount of creep deformation. The main creep mechanism of the single crystal of LiTaO_3_ was suggested to be the dislocation motion [2].

Electrical discharge machining of ceramic composites based on yttria-stabilized tetragonal zirconia is often complicated because of their high electrical resistivity, high hardness, and low fracture toughness, as well as their insufficient electrical conductivity. Nanosized TiN reinforcing particles can be added to produce electrically conductive ceramic nanocomposites that are suitable for electrical discharge machining using two different techniques: either by admixing a commercially available TiN nanopowder synthesized by mechanochemical processing (30 vol% TiN), or 15 vol% of TiN synthesized by the forced hydrolysis of titanium oxysulphate solution (in situ synthesis) and the direct nitriding of the synthesized titanium-oxide nanoparticles [3].

To lower waste disposal and manufacturing costs, waste alumina powder generated by green body machining may be used as a substitute for a certain amount of virgin ceramic powder. It has been shown that 20 wt.% of waste alumina powder may be used to successfully prepare a stable 70 wt.% (approximately 40 vol.%) alumina–waste alumina aqueous suspension for slip casting. Based on the minimal apparent viscosity, an optimum weight content of different additives has been proposed including dispersant Tiron, binder poly (vinyl alcohol), and magnesium aluminate spinel, which inhibits abnormal grain growth. This experiment was based on the Box–Behnken three-factorial response surface methodology. The zeta potential measurements confirmed the stability of the suspension at different pH values [4].

The non-linear problem of the mechanical stability of functionally graded materials which gradually change from 100% Al to 100% Al_2_O_3_, and have thicknesses between 1 and 1.8 mm, was studied using the semi-analytical method (SAM) based on non-linear Koiter’s theory, and with the finite element method (FEM). It was found that the influence of the number of layers increasing from 5 to 15 on the plate stability was negligible. When two applied modeling methods were compared, they showed a good level of agreement. The layer’s thickness did not influence the post-buckling paths. When analyzed with FEM, the plates deflect earlier than during SAM analysis. After the critical load, the SAM analysis showed a larger deflection compared to FEM [5].

In modern-day applications, ceramics are often used as coatings on metals, thus allowing engineering components to be more malleable compared to purely ceramic parts yet more corrosion-, heat-, and wear-resistant than uncoated metals. Good interface relationships between ceramic coatings and metallic base materials are crucial for the design of structural components. A study into the use of alumina films on a nickel substrate focused on the interface damage evolution and fracture properties of the interface using a three-dimensional finite element model. The dependency of the interface fracture energy and interface strength on the residual stress and the thickness of the films was studied. The interface strength increased significantly when a nano-scale film grain size was modeled. The interface strength decreased with an increase in axial residual stress and increased with radial residual stress. The interface strength was not affected by the film thickness, but the interface damage rate increased significantly, which may lead to catastrophic consequences in practice. The underlying mechanisms of different interface damage rates were also assessed [6].

A TiO_2_ photocatalytic film immobilized on porous alumina was prepared using a combined replica and sol–gel method. It was found that, compared to suspended TiO_2_ nanoparticles, immobilized TiO_2_ had a slightly lower efficacy, yet it was still very effective in removing memantine, a pharmaceutical micropollutant, from the water. Immobilized and suspended TiO_2_ had half-lives for memantine degradation of 13.73 min vs. 8.99 min, and the percentage of photocatalytic memantine degradation after 60 min was 96% vs. 99%. The results indicate that using a TiO_2_-coated alumina foam composite material would be much more practical than using TiO_2_ suspensions, which require nanoparticle separation after photocatalytic degradation while keeping the efficacy at an appropriate level [7].

Research on advanced technical ceramics is certainly of great interest to modern industry. With their unique properties, advanced technical ceramics are now crucial materials in various sectors, ranging from consumer electronics, environmental protection, and healthcare to engineering. On 3 October 2023, the European Commission adopted the Recommendations on 10 critical technology areas to enhance the European Union’s economic security [8]. In the areas of Advanced Materials, Manufacturing, and Recycling Technologies, alongside other clean and resource-efficient technologies, there are technologies which support the use of advanced ceramic materials that have been explicitly indicated as being relevant. To support industry and boost investments in critical technologies, on 29 February 2024, the Strategic Technologies for Europe Platform (STEP) was set up by the European Union [9]. This platform will facilitate further research and development, and long-term competitiveness, for advanced ceramic materials and technologies.

We hope that this Special Issue will drive the curiosity of fellow scientists, engineers, students, and the public and inspire and incentivize them to contribute to the study of advanced ceramic materials.
